# Improving the Performance of the Prony Method Using a Wavelet Domain Filter for MRI Denoising

**DOI:** 10.1155/2014/810680

**Published:** 2014-04-14

**Authors:** Rodney Jaramillo, Marianela Lentini, Marco Paluszny

**Affiliations:** Escuela de Matemáticas, Facultad de Ciencias, Universidad Nacional de Colombia, Medellín, Colombia

## Abstract

The Prony methods are used for exponential fitting. We use a variant of the Prony method for abnormal brain tissue detection in sequences of *T*_2_ weighted magnetic resonance images. Here, MR images are considered to be affected only by Rician noise, and a new wavelet domain bilateral filtering process is implemented to reduce the noise in the images. This filter is a modification of Kazubek's algorithm and we use synthetic images to show the ability of the new procedure to suppress noise and compare its performance with respect to the original filter, using quantitative and qualitative criteria. The tissue classification process is illustrated using a real sequence of *T*_2_ MR images, and the filter is applied to each image before using the variant of the Prony method.

## 1. Introduction

The main goal of this paper is to detect abnormal tissues in *T*_2_ weighted magnetic resonance image sequences. The identification of the distribution of relaxation times in *T*_2_ weighted magnetic resonance brain images has been used to classify tissues and as a tool for the segmentation of tumors [1–3]. This is possible because the tissue corresponding to a given pixel in a sequence of *T*_2_ weighted MRI images is characterized by a relaxation rate and therefore allows for differentiation of the tissues that conform to the region determined by a set of pixels. In this paper, we assume that the intensity at a pixel, in a sequence of *T*_2_ weighted MRI brain images, can be approximated by

(1) $$\begin{array}{c} y_{i}=b+\overset{k}{\underset{j=1}{\sum }}C_{j}e^{-i\lambda_{j}\Delta t},\;i=1,\ldots ,n, \end{array}$$

where Δ*t* is the echo time interval. Here, *y*_*i*_ represents the intensity of gray in the pixel, *n* is the number of images in the sequence, and *k* is the number of tissues considered in the pixel, *n* ≥ 2*k* + 1. The exponents *λ*_*j*_ are called the nonlinear parameters and correspond to the relaxation rates associated with the different tissues present in the images, and the coefficients *b*, *C*_1_,…, *C*_*k*_ are called the linear parameters and are related to the background noise of the image and the proportions of each one of the *k* tissues in the pixel, respectively. The background noise is the noise which comes from all possible sources, some of which are unknown, and usually corresponds to patient movement and the image acquisition process. Martín-Landrove et al. [3, 4] proposed the model and used a variant of Prony's method to find the linear and nonlinear parameters. We have used two methods to compute the parameters in the exponential model (1): the variant of the Prony method already mentioned [3–6] and the variable projection method of Golub and Pereyra [7–9]. Prony-type methods are widely used for estimating a signal frequency component; a discussion of such methods can be found in [10–13]. On the other hand, the variable projection method has been used in many applications in fields like electrical engineering, medical image processing, chemical sciences, environmental sciences, magnetic resonance applications, and so forth. A comprehensive bibliographic reference can be found in [8], (144 items).

In [5] Paluszny et al., comparing the solution obtained by the variant of the Prony method and the solution given by the variable projection method, it was noted that both methods successfully converge in the case of low level noisy data, and none produces a satisfactory solution for high noise levels; the variable projection method proved to be more robust in the presence of higher noise levels but required ten times more computational time to get results comparable to those of the variant of the Prony method.

The exponential fit given by (1) is an ill-conditioned problem, and this means that relatively small changes in the data may result in large variations in the linear and nonlinear parameters; therefore, a filter is required to modify the data before using any of the considered methods. In particular, [6] provides an analysis of the sources of instability for the variant of the Prony method.

In the literature, there are a number of different filters to enhance MRI. We chose to modify the filter designed by Kazubek [14], to get a wavelet domain filter for removing noise on each image, before applying the Prony method, and trying to keep the computation time low.

## 2. Wavelet Domain Filters for MRI Denoising

### 2.1. The Two-Dimensional Wavelet Transform

Let us consider an image *f*, and let *f*(*x*, *y*) be the gray intensity corresponding to the pixel given by coordinates (*x*, *y*). The discrete wavelet transform (DWT) is a decomposition of *f* in two functions:

(2) $$\begin{array}{c} f\left(x,y\right)\approx F_{s}\left(x,y\right)+F_{w}\left(x,y\right), \end{array}$$

where **F**_*s*_ is a representation of *f* in terms of shifted and dilated versions of a low-pass scaling function *ϕ*(*x*, *y*) and **F**_*w*_ is a representation of *f* in terms of shifted and dilated versions of a prototype bandpass wavelet function *ψ*(*x*, *y*). The coefficients which determine the components **F**_*s*_ and **F**_*w*_ are called scaling coefficients and wavelet coefficients, respectively. Once the functions *ϕ*(*x*, *y*) and *ψ*(*x*, *y*) have been chosen, the components **F**_*s*_ and **F**_*w*_ are uniquely determined by the coefficients; therefore, it is said that the discrete wavelet transform consists of the two sets of coefficients. The scaling coefficients allow for a low resolution reconstruction of the image and the wavelet coefficients deal with the reconstruction of the details.

There is a large amount of bibliographic information about the theoretical as well as computational aspects of the DWT; we will give, here, a brief description to point out the relevant issues regarding the design of a filter, in the wavelet domain, for MR images.

### 2.2. Wavelet Domain Filters for MRI

When we consider a noisy image and its DWT, it is supposed that the noise in the image is determined by the noise associated with the scaling and wavelet coefficients. A wavelet domain filter corresponds to two procedures, one for the noise reduction in the scaling coefficients and the other for dealing with the noise associated with the wavelet coefficients. The filtered image is the result of applying the inverse wavelet transform to the modified coefficients. The differences between various proposals for wavelet domain filters lie in the algorithms to be applied to each one of the two sets of coefficients; see, for example, [14–22].

Although the noise in MRI comes from various sources, we consider that the perturbation in magnitude follows a Rice distribution, [15, 23]. Kazubek considers that most of the energy used in the image generation corresponds to the scaling coefficients and hence these contribute the most to the perturbation of the image in the presence of noise; therefore, Kazubek proposes to apply a filter to reduce the noise in the scaling coefficients under the hypothesis that the noise is Rician.

According to some authors, numerical experiments show that noise in the wavelet coefficients can be approximated by a Gaussian distribution; for this reason we made a satisfactory noise reduction in these coefficients using a Wiener filter (consult [14, 15, 21]) in addition to the Kazubek filter in the scaling coefficients.

The arithmetic mean of a random variable which follows a Rician distribution is a biased estimator of the value without noise. The next section shows a brief description of the Rician noise and a formula for reducing the bias between the value without noise and the arithmetic mean. We will use this formula later in the filter, on the scaling coefficients.

## 3. Removing the Bias from Rice Distributed Data

In a magnetic resonance image, the gray intensity corresponding to a given pixel is the magnitude of a complex valued function whose real and imaginary components are distributed as Gaussians with the same standard deviation. The different approaches for MRI noise removal can be divided into three classes; some methods act on the real and imaginary components, most of the methods deal with the square of the magnitude, and a third kind of methods acts on the magnitude of the complex image. We are interested in the latter class of methods because our data are in magnitude.

In [14], Kazubek implements a wavelet domain filter which acts on the magnitude of the image and whose relevant issues are the use of the Haar system of orthogonal functions in the wavelet transform and a formula to apply on the scaling coefficients that is used to suppress the bias discussed in Section 2.

Next, we present an analysis of Kazubek's formula for bias removal and propose a modification of his algorithm by applying the bilateral filter to the scaling coefficients [24].

Let *A*_1_ and *A*_2_ be the mean values of two random variables *μ*_1_ and *μ*_2_, respectively, which are the real and imaginary components of a complex image. If *μ*_1_ and *μ*_2_ are distributed as Gaussians with standard deviation *σ*, then the probability density function for the magnitude, $\mu =\sqrt{\mu_{1}^{2}+\mu_{2}^{2}}$, is a Rice distribution given by

(3) $$\begin{array}{c} p_{\mu }\left(\mu \;|\;A\right)=\frac{\mu }{\sigma^{2}}e^{-(\left(\mu^{2}+A^{2}\right)/2\sigma^{2})}I_{0}\left(\frac{A\mu }{\sigma^{2}}\right), \end{array}$$

where *I*_0_ is the modified Bessel function of the first kind and zeroth order and $A=\sqrt{A_{1}^{2}+A_{2}^{2}}$ is the underlying noise-free signal amplitude. Let *m* be the mean value of *μ*; then,

(4) mσ=v(x)∶=π2[(1+x22)I0(x24)+x22I1(x24)]e−x2/4,

where *x* = *A*/*σ* and *I*_1_ is the modified Bessel function of the first kind and first order [15].

Let *z* = *m*/*σ*; we can get a numerical approximation of the inverse *x* = *v* ^−1^(*z*) for 0 ≤ *x* ≤ 50.

We have that *x* ∈ (0, 50] implies −*∞* < 20 log⁡_10_ *x* < 20 log⁡_10_50 ≈ 33.98; then, the inverse function *v* ^−1^ corresponds to a signal whose noise measurement runs from −*∞* to 34 decibels, (dB).

Bessel functions *I*_0_ and *I*_1_ satisfy *I*_0_′ = *I*_1_; then,

(5) v′(x)=π2e−x2/4[3x2I1(x24)+x2I0(x24)+x34I1′(x24)−x34I0(x24)]

and using the power series expansion of functions *I*_0_, *I*_1_, and *I*_1_′, we get

(6) v′(x)=π2e−x2/4[∑k=0∞x4k+12(64k)(k!)2+∑k=0∞x4k+316(k+1)(64k)(k!)2].

(Details of the proof can be found in the Appendix.) Therefore, *v*(*x*) is a monotonically increasing function in the interval [0, +*∞*) and *v*′(0) = 0.

Using polynomial approximations of the functions

(7) $$\begin{array}{c} \frac{x^{2}}{4}I_{0}\left(\frac{x^{2}}{4}\right)e^{-x^{2}/4},\;\;\frac{x^{2}}{4}I_{1}\left(\frac{x^{2}}{4}\right)e^{-x^{2}/4} \end{array}$$

for $x>\sqrt{15}$ (consult Abramowitz and Stegun [25]), we get

(8) $$\begin{array}{c} \underset{x\to \infty }{\lim}\frac{v\left(x\right)}{x}=L, \end{array}$$

where

(9) $$\begin{array}{c} L=0.999999+Er \end{array}$$

and |*Er*| ≤ 5.139(10^−7^). Then, for large *x*, *v*(*x*) behaves as the function

(10) $$\begin{array}{c} H\left(x\right)=\sqrt{L^{2}x^{2}+v{\left(0\right)}^{2}}. \end{array}$$

On the other hand, we have

(11) $$\begin{array}{c} H\left(0\right)=v\left(0\right),\;\;H^{\prime }\left(0\right)=v^{\prime }\left(0\right)=0,\\ \underset{x\to \infty }{\lim}\frac{H\left(x\right)}{x}=L,\;H^{\prime }\left(x\right)>0,\;\forall x>0, \end{array}$$

and a straightforward computation shows that

(12) $$\begin{array}{c} H^{-1}\left(z\right)=\sqrt{\frac{z^{2}}{L^{2}}-\frac{v{\left(0\right)}^{2}}{L^{2}}}. \end{array}$$

This function is a particular case of function $\sqrt{az^{2}+b}$ with *a* > 0 and *b* < 0.

The idea to get a numerical approximation of *v* ^−1^(*z*) consists in considering a function Φ(*z*) such that function

(13) $$\begin{array}{c} F\left(z\right)=\sqrt{az^{2}+b+\Phi \left(z\right)} \end{array}$$

and its derivative satisfy similar asymptotic features of *v* ^−1^(*z*) and [*v* ^−1^]′(*z*), respectively. Let *c* < 0 and let *d* < 0; if we consider Φ(*z*) = *ce* ^*dz*^, then

(14) $$\begin{array}{c} F^{\prime }\left(z\right)>0,\;\;\underset{z\to \infty }{\lim}\frac{F\left(z\right)}{z}=\sqrt{a}<\infty . \end{array}$$

*H*_1_(*z*) = *az* ^2^ is a monotonically increasing function in the interval [0, *∞*) with *H*_1_(0) = 0. Function *H*_2_(*z*) = −*b* − *ce* ^*dz*^ takes positive values and is a monotonically decreasing function in the same interval. Then, there is a unique positive root, *z*_0_, of

(15) $$\begin{array}{c} az^{2}=-\left(b+ce^{dz}\right) \end{array}$$

and if *z* ≥ *z*_0_, then

(16) az2+b+cedz≥az02+b+cedz≥az02+b+cedz0=0.

It follows that [*z*_0_, *∞*) is the domain of

(17) $$\begin{array}{c} F\left(z\right)=\sqrt{az^{2}+b+ce^{dz}},\;\;F\left(z_{0}\right)=0,\\ \underset{z\to z_{0^{+}}}{\lim}F^{\prime }\left(z\right)=\infty . \end{array}$$

**Figure 1:**
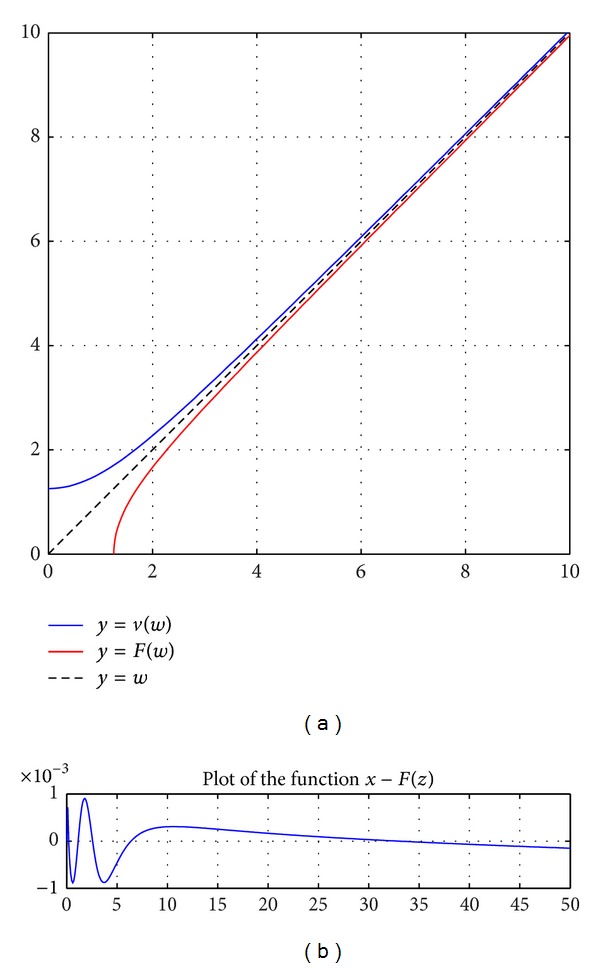
(a) shows the function *F* as a numerical approximation of *v* ^−1^. (b) is a plot of the function *x*_*j*_ − *F*(*z*_*j*_).

To get the parameters *a*, *b*, *c*, and *d*, we perform a least square fit to the discretized curve *F*(*z*) for *x*_*j*_ = *j*(0.1) and *z*_*j*_ = *v*(*x*_*j*_), *j* = 1,…, 500. This problem was solved numerically using the MATLAB function* lsqcurvefit*. With MATLAB R2008a and taking the initial values [*a*_0_, *b*_0_, *c*_0_, *d*_0_] = [1, −1, −2, −1], we get the optimal solution: *a* = 1.0000108, *b* = −1.0122372, *c* = −2.7102422, and *d* = −1.2598921.  Figure 1(a) shows the function *F*(*z*) as a numerical approximation of *V* ^−1^(*z*) and Figure 1(b) shows the quality if the solution given by the MATLAB function* lsqcurvefit*. In MRI, as in acoustic, electricity, and telecommunications, the signal to noise ratio (SNR) is frequently used to measure, in decibels, the noise impact on a signal. Let *r* and *t* be two gray level images, a reference image *r*(*x*, *y*) and a noisy image *t*(*x*, *y*), of size *n*_*x*_ *n*_*y*_. The SNR is computed as

(18) $$\begin{array}{c} \text{SNR}=10{\;\log}_{10}\left(\frac{\sum_{1}^{n_{x}}\sum_{1}^{n_{y}}{\left[r\left(x,y\right)\right]}^{2}}{\sum_{1}^{n_{x}}\sum_{1}^{n_{y}}{\left[r\left(x,y\right)-t\left(x,y\right)\right]}^{2}}\right). \end{array}$$

Nowak [15] suggests that a SNR greater than 10 dB corresponds to a noisy image, whose Rice distribution can be approximated by a Gaussian distribution. We will consider a method to suppress the bias in our MR images using the inverse function discussed in this section when SNR is less than 34 dB and a simple wavelet domain filter for the other cases.

## 4. Implementing a New Wavelet Domain Bilateral Filter for MRI

### 4.1. Formula for the Scaling Coefficients

The component **F**_*s*_(*x*, *y*) in (2) is a linear combination of functions that belong to a specific family of orthogonal functions; each scaling coefficient depends on three parameters: *L*, the level of decomposition in the discrete wavelet transform, and the pair (*k*_1_, *k*_2_) which characterizes the support of the corresponding orthogonal function. Let *f*(*x*, *y*) be the function in (2); if we apply the discrete wavelet transform using the Haar system of orthogonal functions, then the scaling coefficients are given by

(19) $$\begin{array}{c} c_{L,k_{1},k_{2}}=\frac{1}{2^{L}}\overset{2^{L}-1}{\underset{i=0}{\sum }}\;\overset{2^{L}-1}{\underset{j=0}{\sum }}f\left(k_{1}2^{L}+i,k_{2}2^{L}+j\right). \end{array}$$

Details can be found in [26].

### 4.2. Wiener Filter for the Wavelet Coefficients

As the scaling coefficients, the wavelet coefficients depend on three parameters *d*_*l*,*h*_1_,*h*_2__, 1 ≤ *l* ≤ *L*. For simplicity, let us write *d* = *d*_*l*,*h*_1_,*h*_2__ and suppose that the noise in the wavelet coefficients may be approximated by a Gaussian distribution [14]. Then, we attenuate the contribution of this coefficient by

(20) $$\begin{array}{c} \hat{d}=\alpha_{d}d,\;0\leq \alpha_{d}\leq 1. \end{array}$$

According to Nowak, we assume that the noise wavelet coefficient is an unbiased estimator of the value of the wavelet coefficient in the noise-free case and denote its mean by *δ* = *E*[*d*]. The filter weight *α*_*d*_ is chosen by minimizing *E*[(*δ* − *α*_*d*_ *d*)^2^]. In fact, if *σ*_*d*_^2^ is the variance of the wavelet coefficient *d*, then

(21) E[(δ−αdd)2]=δ2−2αdδ2+αd2E[d2]=δ2−2αdδ2+αd2(σd2+δ2);

therefore *α*_*d*_ is defined by

(22) $$\begin{array}{c} \alpha_{d}=\frac{\delta^{2}}{\delta^{2}+\alpha_{d}^{2}}. \end{array}$$

Let *σ*_∗_^2^ be an estimate of *σ*_*d*_^2^; then,

(23) $$\begin{array}{c} \alpha_{d}\approx \alpha_{*}=\frac{E\left[d^{2}\right]-\sigma_{*}^{2}}{E\left[d^{2}\right]}. \end{array}$$

Usually the parameter *σ*_∗_^2^ is approximated by *τσ* ^2^, where *τ* ≥ 1 is a new parameter to be chosen for each image and *σ* is as defined in Section 2. In previous works [15, 18, 21], the value *τ* = 2 has proven to be convenient. Hence, the filter weight in (20) is defined by

(24) αd=(E[d2]−2σ2E[d2])+={E[d2]−2σ2E[d2],if  E[d2]>2σ2,0,otherwise.

### 4.3. Bilateral Filtering

The bilateral filter is a nonlinear filter for images, proposed by Tomasi and Manduchi [24], whereby contours are successfully recovered from noisy images. Let **x** ∈ *I* be a pixel in image *I*; the bilateral filter takes a sum with weights on the pixels in a local neighborhood of **x**, *N*_**x**_. These weights depend on the spatial distance and the intensity in each pixel in the neighborhood. The response of the bilateral filter in the pixel is given by

(25) $$\begin{array}{c} \tilde{I}\left(x\right)=\frac{1}{C}\underset{y\in N_{x}}{\sum }W_{S}\left(x,y\right)W_{R}\left(x,y\right)I\left(y\right), \end{array}$$

where

(26) WS(x,y)=exp⁡−||x−y||22ρd2,WR(x,y)=exp⁡−|I(x)−I(y)|22ρr2,C=∑y∈NxWS(x,y)WR(x,y).

The performance of the bilateral filter depends on the choice of *ρ*_*d*_, *ρ*_*r*_ and the size of the neighborhood *N*_**x**_. Let **x** be a pixel located close to an edge which separates two regions of the image. When the pixel **y** is in the same region as **x**, *W*_*R*_(**x**, **y**) is close to one; on the other hand, when **y** is in the other region, then *W*_*R*_(**x**, **y**) is close to zero if the intensities *I*(**x**) and *I*(**y**) differ greatly and then the filter tends to preserve the pixel intensity due to the effect of the *W*_*R*_(**x**, **y**) components.

In our proposal the bilateral filter is applied to the two-dimensional array consisting of the scaling coefficients of the wavelet transform *I*(**k**) = *I*(*k*_1_, *k*_2_) = *c*_*L*,*k*_1_,*k*_2__. We follow Anand and Sahambi [20] and choose the neighborhood *N*_**x**_ to be a 15 × 15 matrix centered at **x** and the parameters *ρ*_*d*_ = 5 and *ρ*_*r*_ = 1.5*σ*, where *σ* is the standard deviation given in Section 3.

### 4.4. Algorithm for MRI Denoising

(1) Compute an approximation of the variance, *σ* ^2^, choosing a rectangular *q*_1_ × *q*_2_ neighborhood *N*_*η*_, in the background of the image [15]:
  (27) $$\begin{array}{c} \sigma^{2}\approx {\tilde{\sigma }}^{2}=\frac{1}{2q_{1}q_{2}}\underset{u\in N_{\eta }}{\sum }{\left[I\left(u\right)\right]}^{2}. \end{array}$$
(2) Perform a level *L* = 3 wavelet decomposition using the Haar system of orthogonal functions.
(3) Perform a bias correction of the level 3 scale coefficients using the inverse function considered in Section 3.
 From (19) we see that
  (28) $$\begin{array}{c} \frac{1}{2^{L}}c_{L,k_{1},k_{2}} \end{array}$$
 is an approximation to the expected value *m* given in (4) so that
  (29) $$\begin{array}{c} z_{N}=\frac{1}{\tilde{\sigma }2^{L}}c_{L,k_{1},k_{2}} \end{array}$$
 is an approximation of the *z* value defined in Section 2. Using (4) and (17), we get
  (30) $$\begin{array}{c} \frac{x}{\tilde{\sigma }}\approx F\left(z_{N}\right). \end{array}$$
 Now we compute the new scaling coefficients ${\bar{c}}_{L,k_{1},k_{2}}$ by
  (31) $$\begin{array}{c} {\bar{c}}_{L,k_{1},k_{2}}=\tilde{\sigma }2^{L}F\left(z_{N}\right). \end{array}$$
(4) Apply the bilateral filter to the two-dimensional array *J*(*k*_1_, *k*_2_), given by the new scaling coefficients: $J(k_{1},k_{2})={\bar{c}}_{L,k_{1},k_{2}}$.
(5) Obtain a provisional denoised image, *I*_*N*_, computing the inverse wavelet transform using the new scaling coefficients and the unchanged wavelet coefficients.
(6) Perform a level *L* = 4 wavelet decomposition of *I*_*N*_ using the Daubechie system with four vanishing moments.
(7) Perform a correction of the wavelet coefficients given in (6) using (20) and (24).
(8) Get a denoised image by computing the inverse wavelet transform on the set of coefficients given by the scaling coefficients in (6) and the new wavelet coefficients in (7).

## 5. Validation of the MRI Filter Applied to Simulated Images

**Figure 2:**
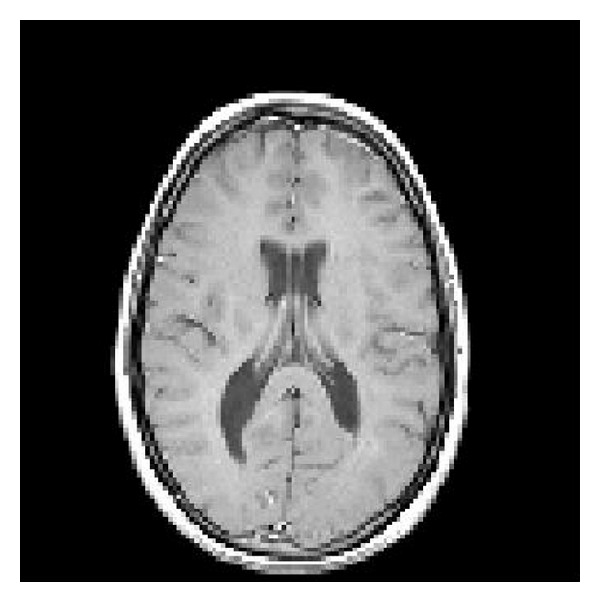
Synthetic image, generated with MATLAB.

We compare the performances of the modified and the original Kazubek's filter using a synthetic image generated with MATLAB, see Figure 2, and introduce the noisy images by adding Rician noise to the synthetic image. The Rician noise was generated as

(32) $$\begin{array}{c} J_{e}\left(m,n\right)=\sqrt{{\left(J\left(m,n\right)+e_{1}\right)}^{2}+e_{2}^{2}}, \end{array}$$

where *J*(*m*, *n*) is the true signal and *e*_1_ and *e*_2_ are random numbers from a Gaussian distribution with mean zero and standard deviation *σ*; five levels of *σ* were used *σ* = [1,2, 5,8, 12], and the gray values in the image are between 0 and 88. To compare the performances of the two filters, we compute five measurements that appear more frequently in the literature: signal to noise ratio (SNR), peak signal to noise ratio (PSNR), root mean square error (RMSE), mean absolute error (MAE), and the structural similarity index (SSIM). Given two gray level images, a reference image *r*(*x*, *y*) and noise image *t*(*x*, *y*), of size *n*_*x*_ *n*_*y*_, the quantities PSNR, RMSE, and MAE are given by

(33) PSNR=10 log⁡10(nxnymax⁡⁡[r(x,y)]2∑1nx∑1ny[r(x,y)−t(x,y)]2),RMSE=1nxny∑1nx∑1ny[r(x,y)−t(x,y)]2,MAE=1nxny∑1nx∑1ny|r(x,y)−t(x,y)|.

The structural similarity index in a region *Ω* is estimated as

(34) $$\begin{array}{c} \text{SSI}{\text{M}}_{\Omega }\left(r,t\right)=\frac{\left(2\mu_{t}\mu_{r}+c_{1}\right)\left(2\theta_{r,t}+c_{2}\right)}{\left({\mu_{r}}^{2}+{\mu_{t}}^{2}+c_{1}\right)\left({\theta_{r}}^{2}+{\theta_{t}}^{2}+c_{2}\right)}, \end{array}$$

where *μ*_*r*_ and *μ*_*t*_ are the means over *Ω* of *r* and *t*, respectively, *θ*_*r*_ and *θ*_*t*_ are the corresponding variances, *θ*_*r*,*t*_ is the covariance of *r* and *t*, and the constants *c*_1_ and *c*_2_ are given by $\sqrt{c_{1}}=0.01*255=2.55$ and $\sqrt{c_{2}}=0.03*255=7.65$; see [27]. If the images are divided in *s* regions, *Ω*_1_,…, *Ω*_*s*_, the SSIM for the two images *r*(*x*, *y*) and *t*(*x*, *y*) is defined by

(35) $$\begin{array}{c} \text{SSIM}=\frac{1}{s}\overset{s}{\underset{j=1}{\sum }}\text{SSI}{\text{M}}_{\Omega_{j}}\left(r,t\right). \end{array}$$

The resultant SSIM index is a value between −1 and 1, and value 1 is only reachable in the case of two identical data sets. Quantities SNR, PSNR, RMSE, and MAE are currently used in computer and medical sciences to compare two images; however, a good quantitative performance could be not consistent, in some cases, with visual perception. SSIM has become a more convenient way to compare two images, when PSNR and RMSE show indistinguishable results for the perception of the human eye.

**Figure 3:**
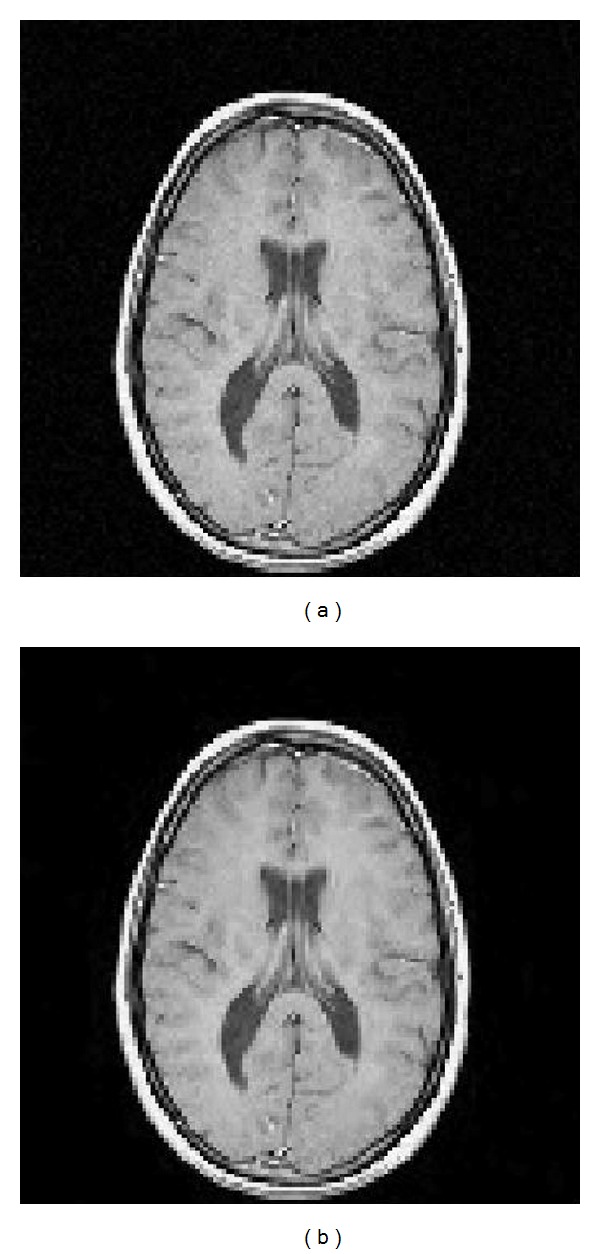
(a) The noisy image corresponding to *σ* = 2. (b) The filtered image using the modification of Kazubek's algorithm.

Tables 1, 2, 3, 4, and 5 show the errors between the noisy images and the filtered images using the quantities SNR, PSNR, RMSE, MAE and SSIM, respectively. The first row in each table corresponds to the noisy image and various *σ* values, the second row corresponds to the filtered image using Kazubek's filter, and the third row corresponds to the filtered image using the modified filter. For each *σ* level, our modified Kazubek's algorithm shows an improved performance with respect to the original algorithm using all the criteria to measure the error. The performance of both filters is nearly indistinguishable in the cases *σ* = 1,2 and we get better filtered images as the noise level increases to *σ* = 5, 8, and 12, see Figure 3.

## 6. Numerical Results

**Figure 4:**
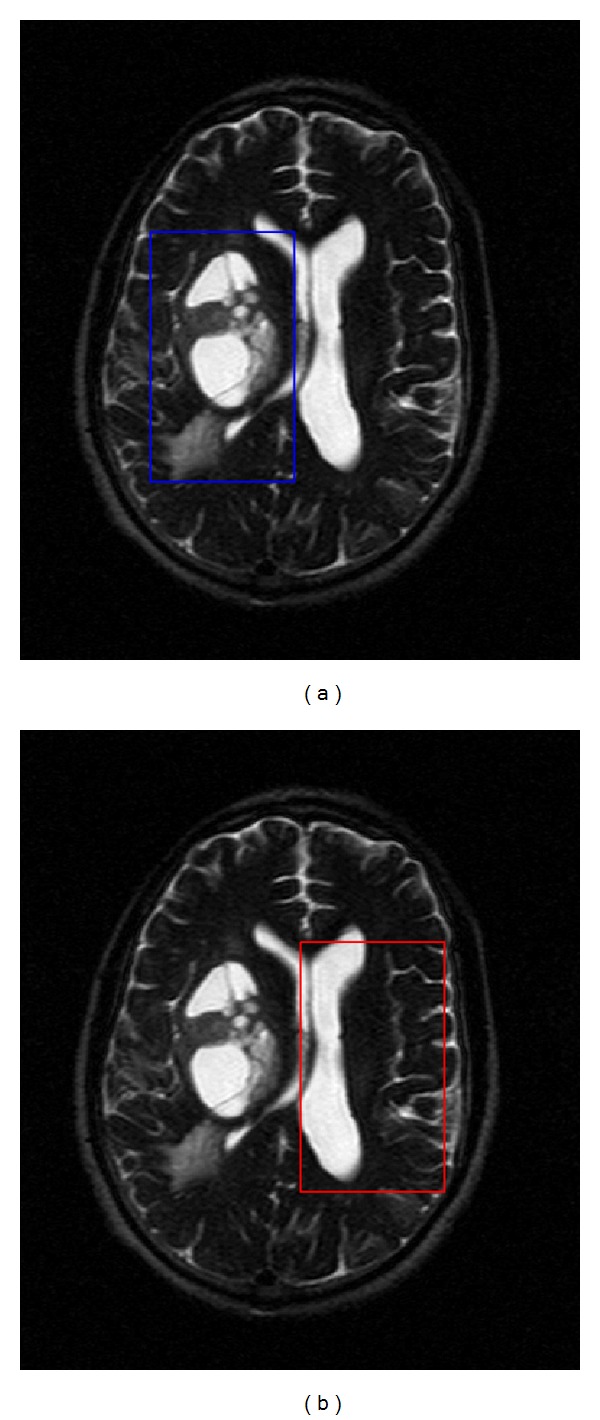
(a) ROI1 and (b) ROI2.

To see the effect introduced by the filter on a real magnetic resonance image, as a part of a tissue segmentation process, we consider the set of images referenced in [3]. In this case, we have a set of eight echoes (magnetic resonance images of the same axial section) from a sequence of T2-MRI, with an echo time of 44 milliseconds. According to [2, 3] we can see a part of the brain affected by tumoral tissues (ROI1) and another region showing no visible impairment (ROI2); regions ROI1 and ROI2 are called the region of lesion and region of control, respectively.  Figure 4 shows the echo corresponding to *n* = 6, from the set of images. For each pixel in a region the variant of Prony method is applied, to find the linear and nonlinear parameters defined in (1) with *n* = 8 and Δ*T* = 0.044; this process is performed for *k* = 1,2, and 3, and we choose the ones minimizing the residual. In Table 6, we show the computation times of the classification process, using our implementation of the variant of the Prony method.

For each region of interest, the frequency diagram is computed as follows: we divided the interval [0, 30] into 100 bins of equal length; **ς**_*j*_ = [*ς*_*j*_, *ς*_*j*+1_], 1 ≤ *j* ≤ 100, since the brain tissues relaxation rates belong to this interval, as we can see in [2]. For the pixels in a specific region and a selected subinterval **ς**_*j*_, we add the linear parameters whose corresponding nonlinear parameters belong to **ς**_*j*_, and this process is done for 1 ≤ *j* ≤ 100, to get a diagram of frequencies. Each frequency diagram is normalized to obtain a probability density function.

**Figure 5:**
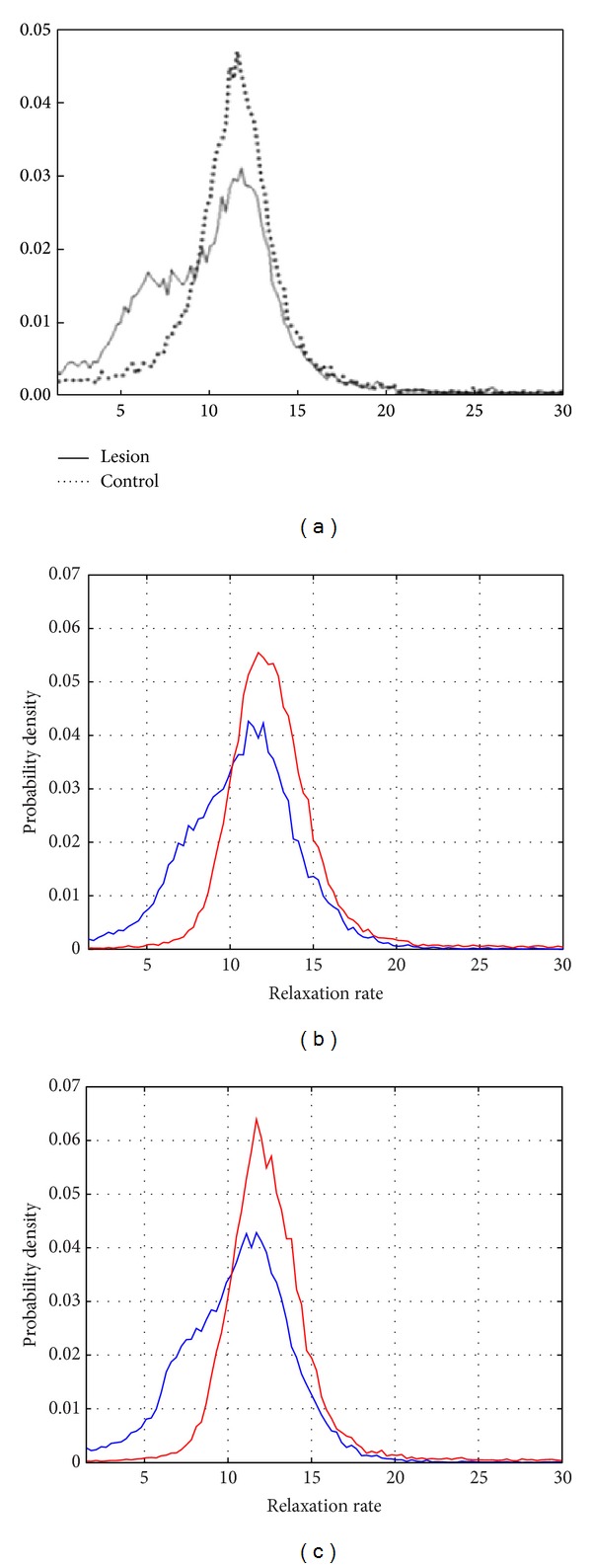
Probability densities corresponding to ROI1 and ROI2, in blue and red, respectively. (a) Graphic reported in [3]. (b) Solution obtained by Prony's method without a filtering process. (c) Solution obtained when we apply the wavelet domain filter to the images before using the Prony method.

In Figure 5(a), we see the solution obtained in [3], which was calculated by the variant of the Prony method.  Figure 5(b) shows the results using our implementation of the variant of the Prony method, in red the probability density corresponding to the control region, and in blue the corresponding to the region of lesion. The differences in the shape of these graphics are caused because our implementation of the method is not the same as that reported in [3]. Figure 5(c) shows the result of applying the MRI filter designed in this paper, on each one of the eight images, before running the variant of the Prony method. The filtering process produces a curve with less dispersion for the region ROI2 and a softer edge in the curve corresponding to region ROI1.

## 7. Conclusion

This paper takes as its starting point the wavelet domain filter for MRI developed by Kazubek. We improved the original filter introducing the bilateral filter in the wavelet domain. The resulting filter exhibits a better performance and requires only a small increase in computational time of the whole tissue classification process. The new filter is applied to real brain MRI in a process of brain tissue classification proposed by Paluszny et al. [3], getting different results to those previously reported, which lead to an improvement in tissue identification with *T*_2_ relaxation techniques. Our results are validated with the main quality criteria for image denoising.

## Figures and Tables

**Table 1:** SNR between the original and the denoised images corresponding to the two filters and *σ* values.

	*σ* = 1	*σ* = 2	*σ* = 5	*σ* = 8	*σ* = 12
Original errors	29.77391	23.7840	16.0170	12.0372	8.9663
Kazubek's algorithm	33.7311	27.9346	20.4575	17.0542	14.1045
Modification to Kazubek's algorithm	34.0501	28.3961	21.4209	18.0140	15.3410

**Table 2:** PSNR between the original and the denoised images corresponding to the two filters and *σ* values.

	*σ* = 1	*σ* = 2	*σ* = 5	*σ* = 8	*σ* = 12
Original errors	37.2277	31.5732	24.4287	21.4391	18.5411
Kazubek's algorithm	41.1923	35.7037	28.6968	25.4961	22.6839
Modification to Kazubek's algorithm	41.5063	36.1647	29.7469	26.9269	24.5610

**Table 3:** RMSE between the original and the denoised images corresponding to the two filters *σ* values.

	*σ* = 1	*σ* = 2	*σ* = 5	*σ* = 8	*σ* = 12
Original errors	1.2495	2.4935	6.1831	10.0105	14.9444
Kazubek's algorithm	0.7914	1.5401	3.6277	5.3316	7.4354
Modification to Kazubek's algorithm	0.7631	1.4618	3.2590	4.8087	6.5236

**Table 4:** MAE between the original and the denoised images corresponding to the two filters and *σ* values.

	*σ* = 1	*σ* = 2	*σ* = 5	*σ* = 8	*σ* = 12
Original errors	1.0522	2.1056	5.2262	8.4441	12.6008
Kazubek's algorithm	0.5491	1.0710	2.4354	3.5928	5.1168
Modification to Kazubek's algorithm	0.5247	1.0195	2.2510	3.3490	4.6331

**Table 5:** SSIM between the original and the denoised images corresponding to the two filters and *σ* values.

	*σ* = 1	*σ* = 2	*σ* = 5	*σ* = 8	*σ* = 12
Original errors	0.9094	0.7705	0.5543	0.4482	0.3640
Kazubek's algorithm	0.9864	0.9567	0.8482	0.7489	0.6532
Modification to Kazubek's algorithm	0.9878	0.9614	0.8636	0.7669	0.6775

**Table 6:** Computation times in seconds corresponding to the tissue classification process.

Method	ROI1	ROI2
Prony without filtering	9.39	9.29
Prony with filtering	11.40	11.29

## Appendix

The power series expansions of functions *I*_0_, *I*_1_, and *I*_1_′ are

(A.1) I0(x)=∑k=0∞x2k4k(k!)2,  I1(x)=12∑k=0∞x2k+14kk!(k+1)!,I1′(x)=12∑k=0∞(2k+1)x2k4kk!(k+1)!.

Therefore,

(A.2) v′(x)=π2e−x2/4[3x2I1(x24)+x2I0(x24)+x34I1′(x24)−x34I0(x24)]=π2e−x2/4[3x4∑k=0∞x4k+243k+1k!(k+1)!+x2∑k=0∞x4k43k(k!)2+x38∑k=0∞(2k+1)x4k43kk!(k+1)!−x34∑k=0∞x4k43k(k!)2]=π2e−x2/4[34∑k=0∞x4k+343k+1k!(k+1)!+12∑k=0∞x4k+143k(k!)2+18∑k=0∞(2k+1)x4k+343kk!(k+1)!−14∑k=0∞x4k+343k(k!)2]=π2e−x2/4[12∑k=0∞x4k+143k(k!)2+∑k=0∞(316(k+1)+2k+18(k+1)−14)×x4k+343k(k!)2]=π2e−x2/4[∑k=0∞x4k+12(64k)(k!)2+∑k=0∞x4k+316(k+1)(64k)(k!)2].

* *
